# An Instrument for *In Situ* Measuring the Volume Scattering Function of Water: Design, Calibration and Primary Experiments

**DOI:** 10.3390/s120404514

**Published:** 2012-04-10

**Authors:** Cai Li, Wenxi Cao, Jing Yu, Tiancun Ke, Guixin Lu, Yuezhong Yang, Chaoying Guo

**Affiliations:** 1 State Key Laboratory of Oceanography in the Tropics, South China Sea Insstitute of Oceanology, Chinese Academy of Sciences, Guangzhou 510301, China; E-Mails: liclaire@scsio.ac.cn (C.L.); ketian@scsio.ac.cn (T.K.); luguixin@scsio.ac.cn (G.L.); wuli@scsio.ac.cn (Y.Y.); guochaoying@scsio.ac.cn (C.G.); 2 South China Sea Fisheries Research Institute, Chinese Academy of Fishery Sciences, Guangzhou 510300, China; E-Mail: yj_scs@163.com

**Keywords:** ocean optics, scattering measurements, array detection, calibration test, in-field measurement, scattering volume, optical radiometric calibration

## Abstract

The optical volume scattering function (VSF) of seawater is a fundamental property used in the calculation of radiative transfer for applications in the study of the upper-ocean heat balance, the photosynthetic productivity of the ocean, and the chemical transformation of photoreactive compounds. A new instrument to simultaneously measure the VSF in seven directions between 20° to 160°, the attenuation coefficient, and the depth of water is presented. The instrument is self-contained and can be automatically controlled by the depth under water. The self-contained data can be easily downloaded by an ultra-short-wave communication system. A calibration test was performed in the laboratory based on precise estimation of the scattering volume and optical radiometric calibration of the detectors. The measurement error of the VSF measurement instrument has been estimated in the laboratory based on the Mie theory, and the average error is less than 12%. The instrument was used to measure and analyze the variation characteristics of the VSF with angle, depth and water quality in Daya Bay for the first time. From these *in situ* data, we have found that the phase functions proposed by Fournier-Forand, measured by Petzold in San Diego Harbor and Sokolov in Black Sea do not fit with our measurements in Daya. These discrepancies could manly due to high proportion of suspended calcium carbonate mineral-like particles with high refractive index in Daya Bay.

## 1. Introduction

The volume scattering function (VSF) *β*(*ψ*) (units of m^−1^sr^−1^), describes the angular distribution of light scattered from an incident unpolarized beam. The VSF and the volume absorption coefficient *α*(*λ*) (m^−1^), provide the most fundamental description of a medium's inherent optical properties (IOPs), as all other IOPs can be derived from them [1–5]. Coupled with the angle and spectral distribution of the incident radiance just below the surface, the full radiative distribution can be simulated based on the radiative transfer equation. All of these simulations can be used to describe the upper-ocean heat balance [3,6], the photosynthetic productivity of the ocean [7–9] and the chemical transmission of photoreactive compounds [10,11]. These simulations are also key to current applications concerning the diagnosis of upper-ocean constituents from the inversion of the spectral distribution of upwelling radiance measured from satellite platforms [12–16].

Despite its fundamental nature, little is known about the variability of the VSF, while the relationship between the absorption property and constituents of water is well known [11,17,18]. This lack of information is largely due to the extreme difficulty of performing direct measurements of the VSF [3,12]. The measurement of the VSF and the scattering coefficients has had a diverse and fragmented history. More than 30 years ago, Tyler [19,20], Kullenberg [21,22] and Petzold [23], among others, developed instruments for measuring the VSF of the ocean across a range of angles. The typical instrument used is the General Angle Scattering Meter designed by Petzold in the 1970s. Most researchers [3,24,25] still base their analyses on a relatively small VSF data set taken in three representative types of seawater offshore Southern California, the San Diego harbor and the tongue of the ocean [23]. All of the instruments historically used for measuring the general-angle VSF of the ocean have a complex mechanical structure because their angular deviation is provided by rotating a bulky light source or photodetector unit around the axis of the scattering volume. These designs and structures result in numerous defects, such as poor repeatability, high power consumption (involving the mechanical rotation of a bulky light source or photodetector unit by motor) and poor data synchronism (with a general measurement taking approximately 30 minutes). In addition, these instruments are complicated and bulky [19–23,26] because of the limitations imposed by their technical constraints. Most of these VSF instruments cannot be used *in situ* to obtain the variation of the VSF with depth, and all data must be obtained from sea water that has been pumped from outside the breaker line and brought in by tank truck to the laboratory [3,20,23]. In the 1980s, Kullenberg [24] used a similar approach and measured the VSF with discrete measurements over a broader angular range (10° to 165°) at two wavelengths (520 nm and 660 nm) in two oceanic areas. Their measurement results indicated that strong forward scattering is due to relatively large suspended particles. They speculated that the magnitude of the volume scattering coefficient was essentially determined by such large particles, and they recorded a broad minimum VSF in the interval of 90 to 120°. In the subsequent twenty years, the development of a general-angle VSF measurement instrument was discontinued. In the 21st century, with the rapid improvement of ocean optics and photoelectric detection techniques, the development of a general-angle VSF measurement instrument was again initiated. There are two representative types of these instruments: the Multi-spectral Volume Scattering Meter MVSM (based on a rotation prism, acquisition time: 1.5 min), which measures the VSF at angles between 0.6∼177.9° [3,27,28], and the Multi-Angle SCattering Optical Tool (MASCOT) (based on the array detection principle, acquisition frequency: 20 Hz), which measures the VSF between 10° and 170° at 10° intervals. In recent years, MVSM and MASCOT have been successfully applied to study the angle variation characteristics of the VSF of the oceanic particulates and oceanic bubbles *in situ* [27–32].

In our study, a new instrument has been developed that can quickly and synchronously measure the attenuation coefficient, the angle distribution characteristics of the VSF, and water depth information in profile *in situ* (profile depth up to 200 m and acquisition frequency of 6 Hz). A calibration method based on the estimation of the scattering volume and optical radiometric calibration is also developed. The variation characteristics of the VSF with angle, depth and water quality in Daya Bay were studied with this instrument for the first time. The VSF measurement principle of this instrument is based on the array detection principle, viewing a common volume to measure the VSF at discrete angles (20°, 50°, 71°, 90°, 126°, 140° and 160°) between 0° to 180° and a specific attenuation coefficient *c* (0°). The instrument is self-contained and automatically controlled by depth underwater, and the data can be easily downloaded via wireless communication (the transmitting distance is no less than 200 m). Here, we present the theoretical background, the instrument design, the calibration method, the experiment in the laboratory and the first VSF observations made by this instrument in Daya Bay in 2010.

## 2. Instrument Developments

**Figure 1.**
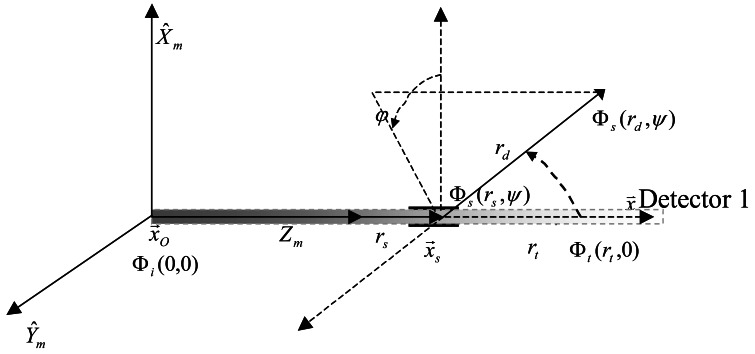
Measuring principle of the VSF.

Scattering causes a fraction of the incident flux to be diverted away from the incident beam at various angles, as shown in Figure 1.

In Figure 1, *φ* is the azimuth angle and *ψ* is the scattering angle, Φ*_i_* (0,0) is the radiometric flux of the collimated source, Φ*_s_* (*r_s_,ψ*) is the scattering flux through scattering volume *V*(*ψ*), Φ*_s_* (*r_d_,ψ*) is the scattering light flux received by the detector, *r_s_* is the distance from the collimated source to the center of the scattering volume, *r_d_* is the distance between the center of the scattering volume and the detector.

In most cases, the scattered light is concentrated in the forward direction, although significant scattering occurs at all angles. Mathematically, the VSF is defined as the second partial derivative of the scattered flux Φ with respect to the solid angle Ω and scattering volume V, normalized by the incident collimated irradiance E, whose unit is m^−1^·sr^−1^, that is [3,23,33]:

(1) $$\beta (\psi )=\frac{\partial^{2}\Phi (\psi ,r_{s})}{E\partial \Omega \partial V(\psi )}$$

where *β*(*ψ*) is the function of the wavelength, although it does not contain the wavelength explicitly. In seawater, *β*(*ψ*) varies in time and space and is generally a function of angle and depth.

The total scattering coefficient *b*(*λ*), backward scattering coefficient *b_b_*(*λ*), and dimensionless scattering phase function *β̄*(*λ, ψ*) can be calculated from the VSF as follows:

(2) $$b=\iint_{4\pi }\beta (\psi )d\Omega =2\pi \int_{0}^{\pi }\beta (\psi )\sin\psi d\psi$$

(3) $$b_{b}=2\pi \int_{\pi /2}^{\pi }\beta (\psi )\sin\psi d\psi$$

(4) $$\tilde{\beta }(\lambda ,\psi )=\frac{\beta (\lambda ,\psi )}{b(\lambda )}$$

The parameters in the mathematical definition of the VSF (Figure 1) can be described as follows:

(5) $$E=\Phi_{i}(0,0)/S\exp(-cr_{s})$$

(6) $$\Phi_{s}(r_{d},\psi )=\Phi_{i}(r_{s},\psi )\exp(-cr_{d})$$

where *S* is the normal cross-sectional area of the collimated exiting light (collimated source), and *c* is the beam attenuation coefficient.

Combining Equations (1), (5) and (6), we can write:

(7) $$\beta (\psi )=\frac{\Phi_{s}(r_{d},\psi )S}{\Phi_{i}(0,0)\exp[-c(r_{s}+r_{d})]\Omega V(\psi )}$$

In Equation (7) *r_s_*, *r_d_*, *V*(*ψ*) and Ω are directly or indirectly computed theoretically from the geometrical parameters of the device, and *β*(*ψ*) can be calculated if the scattering light flux Φ*_s_* (*r_d_,ψ*) and attenuation coefficient *c* are obtained. In the next sections, we discuss how this method can be implemented.

### 2.1. Optical and Mechanical Structure Design

#### 2.1.1. Light Source

**Figure 2.**
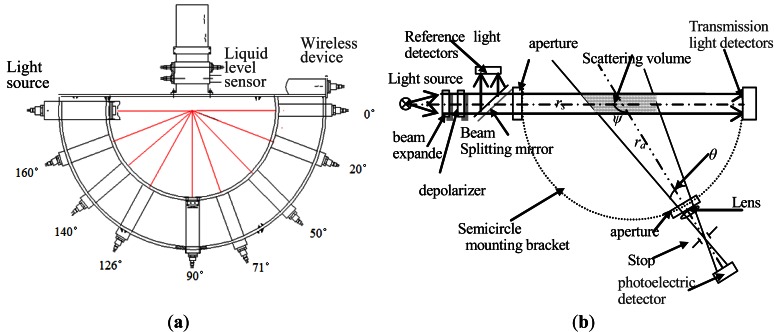
(**a**) Architecture of the array general-angle volume scattering function measuring instrument; (**b**) Optical structure of the measuring instrument (light source and detector).

A 650 ± 5 nm semiconductor laser, which provides approximately 50 mW peak power output, is mounted on a set of miniature positioning stages so its alignment can be precisely adjusted (Figure 2). The laser passes through a beam expander that enlarges it to 10 mm and reduces the beam divergence to less than 0.02 mrad, generating a collimated beam. Because scattering is sensitive to polarization, the laser beam is directed through a depolarizer (composed of linear polarizing film and a quarter-wave plate). While the depolarizer does not truly depolarize the light in the sense of transforming it into circularly polarized light, it does “scramble” the beam such that it includes a continuous range of polarization states that are evenly distributed throughout the beam cross-section. After depolarization, the beam enters a half-transparent and half-reflective mirror that reflects 10% of the beam to a reference detector while 90% of the beam is transmitted to the plane of the aperture and is output through the optical glass as the light source of instrument Φ*_i_* (0,0). The output signal of the reference detector is used to normalize the measured signals and compensate for variation in the laser's output. Using an oscillating circuit, the laser is modulated at 400 Hz, and the transmission and scattering receivers are synchronized to the modulating signal to distinguish between scattered light and the solar background. The above structure arrangement and work light source selection ensures the instrument structure's shading effect do not exist in this instrument at all.

In Figure 2(a), The detectors and light sources are coplanar, and the angles (ψ) between them are 0, 20, 50, 71, 90, 126, 140 and 160°, respectively. A wireless device is fixed on the top of the semicircle rack, and a liquid level sensor is configured to measuring the underwater profile depth. In Figure 2(b), ψ is the scattering angle and θ is the angle of half field-of view (FOV).

#### 2.1.2. Receivers

Eight receivers for simultaneously measuring beam transmission and scattering at seven discrete angles are mounted on a semicircle with a diameter of 50 cm (Figure 2(a)). The detectors and light sources are coplanar, and the angles (*ψ*) between them are 0, 20, 50, 71, 90, 126, 140 and 160°, respectively. These scattering angles were selected because they are very optimal angles for study the relationship between the scattering property and constituents of water. For example, bubble scattering in water can be explained by the ratio of VSF near 70° to VSF near 120°, scattering between 120° to 160° are very important and most controversial in estimating backscattering coefficient, 90° is a most critical angle for study the VSF of water, scattering near 45° is proportional to scattering coefficient, and with 0° transmission, the attenuation coefficient and VSF can be determined completely by this instrument. Each scattering receiver has an interference filter to reduce solar background, a lens with a 10 cm focal length, and a 3.2 mm diameter stop mounted on the focus to reduce its response to extraneous light. Each scattering receiver has a field of view half-angle of 0.92°.

Because the amount of scattering varies drastically depending on the angle and because the scattering signals are relatively large in forward angles and extremely small in the angles greater than 70°, the preamplifier electronics in the receivers have discrete, electronically controlled gain settings to adapt to the different scattering levels.

**Figure 3.**
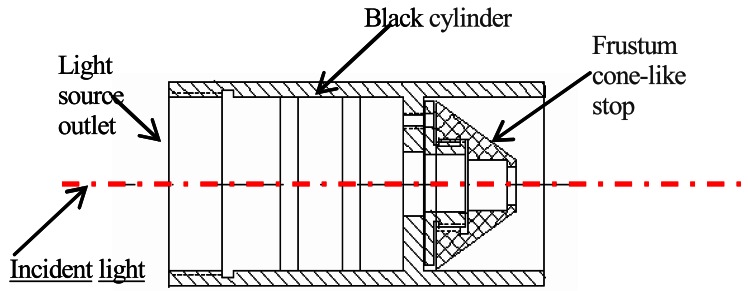
The equipment for reducing the reflected light from transmission receiver.

**Figure 4.**
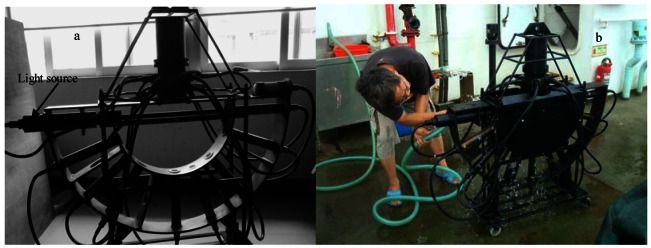
(**a**) Light source and receiver array of the VSF instrument; (**b**) The actual structure with two light-tight plates on each side of the semicircle.

Figure 2 only shows a theoretical configuration. In fact, to minimize the reflection and thoroughly eliminate solar background, the following improvements are made in the light source and receivers:

1. To reduce the reflected light flux from the transmission receiver, a Teflon film was used to cover the photoelectric detector in the transmission receiver to change the specular reflectance to diffuse reflectance; the photoelectric detector was also moved as close as possible to the stop to improve the sensitivity of the response.
2. The equipment shown in Figure 3 was mounted in front of the outlet of the light source; the equipment mainly consists of a black cylinder and a frustum cone-like stop. During the light transmission process, the reflection light returns to this equipment. Only part of reflected light can be transmitted to the light source, with the rest reflected back and forth between the outside of the frustum cone-like stop and inside the black cylinder. The actual light source is shown in Figure 4(a).
3. To eliminate the influence of the solar background on the scattered light signal more thoroughly, two light-tight plates were mounted on each side of the semicircle (Figure 4(b)).

**Figure 5.**
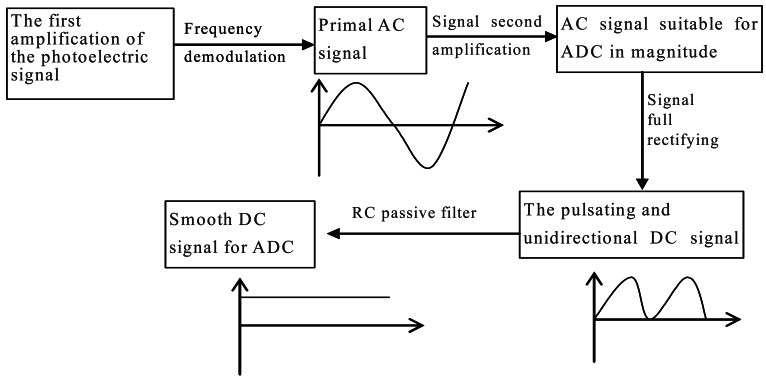
Flow diagram of the signal process.

The received photoelectric signal must be demodulated and amplified to distinguish the primal signal from the solar background to be suitable for the analog to digital converter (ADC). Signal processing mainly includes signal amplification, frequency demodulation, full wave rectification and filtering. Figure 5 is a flow diagram of the signal process.

In this figure AC, DC, A/D, and RC mean current, direct current, analog to digital, resistance/capacitance, respectively.

Here, we illustrate the procedure of signal processing shown in Figure 5. The signals received by the transmission and scattering sensors are weak and originate from various directions. Therefore, before frequency demodulation, preamplification must be performed to amplify the signals to a level high enough to distinguish the primal signals from the background noise through a second-order active band pass filter (*i.e.*, frequency demodulation in Figure 5 with a center frequency of 400 Hz). During frequency demodulation, the primal AC signals are amplified concurrently to a level suitable for A/D conversion, and then a full rectifying and RC passive filter are joined to make the AC signal a smooth DC signal just for the ADC.

#### 2.1.3. Sampling Process Control and Data Download

**Figure 6.**
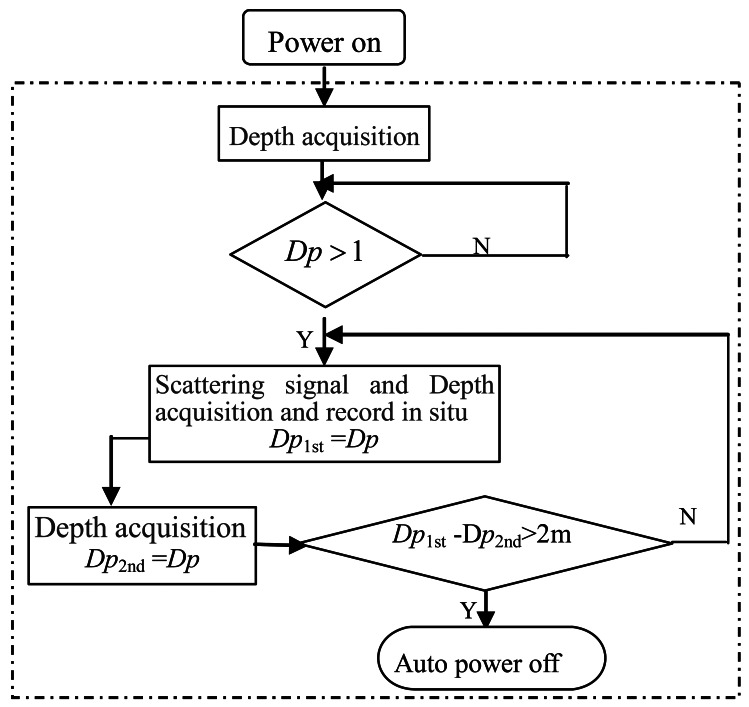
Flow diagram of the automatically control processing under water.

The instrument is a self-contained system, and it can be automatically controlled by the underwater depth. The sampling process cannot begin until the instrument has been completely immersed in water, and will continue if the depth increases and stop if the depth decreases. In other words, the sampling process is unidirectional, and it only occurs while the instrument is lowered through the water. Low power consumption and high data availability can be achieved with this control method. Figure 6 is a flow diagram of the automatically control processing of this instrument system underwater.

In Figure 6, *Dp* means the current profile depth of the instrument, *Dp_1st_* and *Dp_2nd_* respectively represent the profile depth of the instrument during two adjacent sampling intervals. There are two ultra-short-wave wireless communication kits: one is located in the instrument (Figure 2), and the other is connected to the serial port of the upper computer. With no connections at all, the self-contained data can be easily downloaded via these two pieces of wireless communication equipment (with a transmitting distance of no less than 200 m). This method of communication is well suited for operating onboard a vessel or on a platform.

### 2.2. Estimation of Scattering Volume

The scattering volume varies with the detection direction of the scattering light: it is smallest at 90° and becomes larger when approaching forward 0° or backward 180°. This is a complex process. Here, we illustrate the calculation method of the scattering volume based on the design of the optical and mechanical structures. In Figures 1 and 2, *r_s_* is the distance from the center of the scattering volume to the light source, *r_d_* is the distance from the scattering receiver to the center of the scattering volume, *θ* is angle of half field-of view (FOV) of the scattering receiver, D is the diameter of the light source aperture, and *ψ* is the scattering angle. Because the beam divergence is less than 0.02 mrad, *r_s_* and *r_d_* are 25 cm, so the divergence of the light source at the distance of *r_s_* can be overlooked and thought of as an even-diameter cylindrical light in the light travel range. The light source distribution is homogeneous, and the receiving FOV at each point of the scattering receiver is equal. In addition, the diameter of the light source is less than the diameter of each scattering receiver. Based on single scattering approximation, the relationship between the scattering volume (including *V*(90°) and *V*(*ψ*)) and the optical and geometrical structures of the instrument can be deducted and computed. Here, we omit the detailed derivation process and only give the derived relationship:

(8) $$V(90{}^{\circ})=\frac{\pi D^{2}tg\theta }{6}\left[2r_{d}+\sqrt{{r_{d}}^{2}-{\left(\frac{1}{2}D\right)}^{2}}\right]$$

(9) $$\begin{array}{rr} V(\psi )=\frac{\pi D^{2}\sin\theta }{6}\frac{\sin\psi \cos\theta }{\sin^{2}\psi -\sin^{2}\theta }\left[2r_{d}+\sqrt{{r_{d}}^{2}-{\left(\frac{1}{2\sin\psi }D\right)}^{2}}\right] & 0{}^{\circ}<\psi <180{}^{\circ}\\ =\frac{\pi D^{2}tg\theta }{6}\frac{\sin\psi \cos^{2}\theta }{\sin^{2}\psi -\sin^{2}\theta }\left[2r_{d}+\sqrt{{r_{d}}^{2}-{\left(\frac{1}{2\sin\psi }D\right)}^{2}}\right] & \end{array}$$

Using the actual values of D, *ψ*, *θ*, and *r_d_* from Equations (8) and (9), we can obtain the estimated result of the scattering volume at arbitrary angle (*V*(*ψ*)) and the scattering volume at 90° (*V*(90°)). We also calculate the *V*(*ψ*) from *V*(90°)/sin (*ψ*), that is:

(10) $$\begin{array}{cc} V(\psi )=V(90)/\sin(\psi ) & 0{}^{\circ}<\psi <180{}^{\circ} \end{array}$$

**Figure 7.**
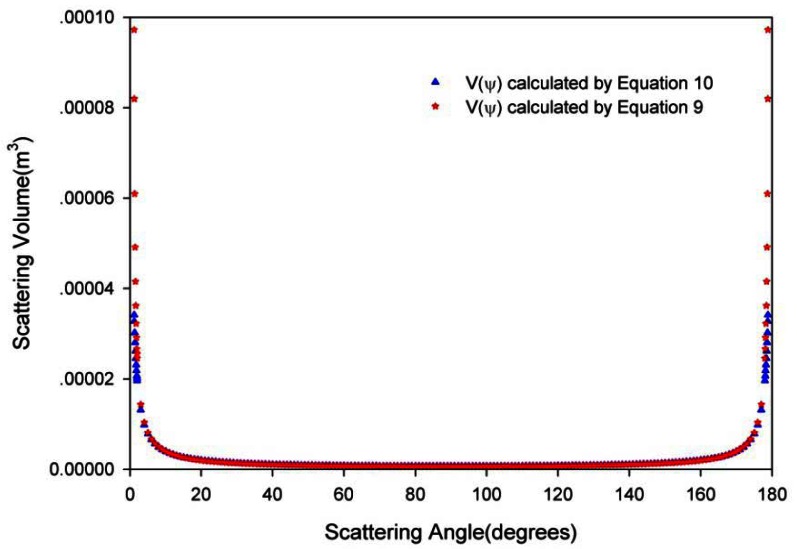
The *V*(*ψ*) calculated from Equations (9) and (10).

Figure 7 shows the *V*(*ψ*) calculated from Equations (9) and (10). The results indicate that Equation (10) can be used to estimate the scattering volume between 20° to 160° and that the calculation error is less than 0.158%; the closer to 90°, the smaller the expected calculation error. When *ψ* tends toward 0° or towards 180°, based on the optical and mechanical structures of instrument (*i.e.*, D, *θ*, and *r_d_*, *etc.*), in Equation (9), *ψ* is finite and the *V*(*ψ*) calculated from it goes to a constant, while *V*(*ψ*) calculated from Equation (10) is infinite. These conclusions correspond well with those of Lee and Lewis [3], Kullenberg [24] and Petzold [23].

The solid angle Ω was calculated from the FOV of the scattering receiver:

(11) Ω=2π(1−cosθ)

Combining Equations (10), (11), we can write Equation (7) as:

(12) $$\beta (\psi )=\frac{\Phi_{s}(r_{d},\psi )SC_{V\Omega }(90^{{}^{\circ}})}{\Phi_{i}(0,0)\exp[-c(r_{s}+r_{d})]}\sin\psi$$

(13) $$C_{v\Omega }\left(90\right)=\overset{1}{\;}/\underset{V(90)\Omega }{\;}=5.11\times 10^{10}m^{-3}sr^{-1}$$

### 2.3. Calibration Test

The *β*(*ψ*) can be calculated from Equation (12) if the relationships are determined between the scattering light flux Φ*_s_*(*r_d_,ψ*) and *DN_s_*(*r_d_,ψ*) and the transmission light flux Φ*_t_*(*r_t_*,0) and *DN_t_*(*r_t_*,0), *DN_s_*(*r_d_,ψ*) and *DN_t_*(*r_t_*,0) are photoelectrical signals received by the scattering light detector and the transmission light detector mounted at (*ψ*, *r_d_*) and (0, *r_t_*).

**Figure 8.**
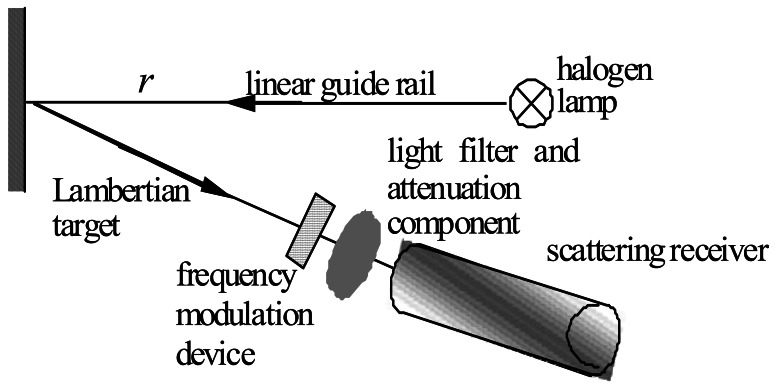
Volume scattering calibration equipment.

In Figure 8, the calibration equipment mainly includes a precision linear guide rail, a Lambertian target, a stable standard light source, a frequency modulation device and a light filter and attenuation component. *r* is the distance between the light source and the Lambertian target.

The scattering receivers were calibrated in an optical laboratory in the air. The length of the guide rail is 3 m, and the frequency modulation device fixed in front of the light filter and the attenuation component is used to modulate the frequency of the light wave received by the scattering sensor. Because the amount of scattering varies drastically depending on the angle, receivers at different locations must be set with a different amplifier gain to be tailored to the dramatically different signal levels. Thus, in this calibration test, the various light attenuation tests must be performed to accommodate the same luminous flux received by each scattering receiver at the same distance. In addition, the halogen lamp must be filtered as a single wavelength only as 650 nm (the working light source wavelength of the instrument). Therefore, the light filter and attenuation component that make up a 650 nm filter and some neutral glass slices are fitted in front of each scattering receiver. Based on the respective amplifier gain and dynamic range of each scattering receiver, the transmissions of the neutral glass slices for the different receivers or different distances are variable.

The stable standard light source is a 1,000 W Quartz halogen spotlighting tungsten lamp (LJS110–1000), which has been calibrated by the National Institute of Measurement and Testing Technology, and measurement equipment used in the verification is referred to in the China National Standards of Measurement. The maximum permissible error is 0.05% (coverage factor *k* = 2).

The calibration method involves measuring the response of each scattering sensor to the Lambertian target as a function of distance *r*. Because the target is Lambertian, the surface scattering function is independent of the scattering angle, and the light flux received by the scattering receivers must be multipliable by *ρ*(650)/*π*. In addition, the light flux received by each scattering receiver must be multiplied by the transmission of the light filter and the attenuation component set in front of the scattering receiver. That is:

(14) $$L\left(650\right)=\frac{\rho \left(650\right)}{\pi \cdot r^{2}}\cdot E_{S}\left(650\right)\cdot T(650)$$

where *ρ*(650) is the diffuse reflectivity of the Lambertian target at 650 nm, *E*_s_(650) is the irradiance of the standard light source in 650 nm, *L*(650) is the radiance in 650, and *T*(650) is the transmission of the light filter and attenuation component in 650 nm.

**Figure 9.**
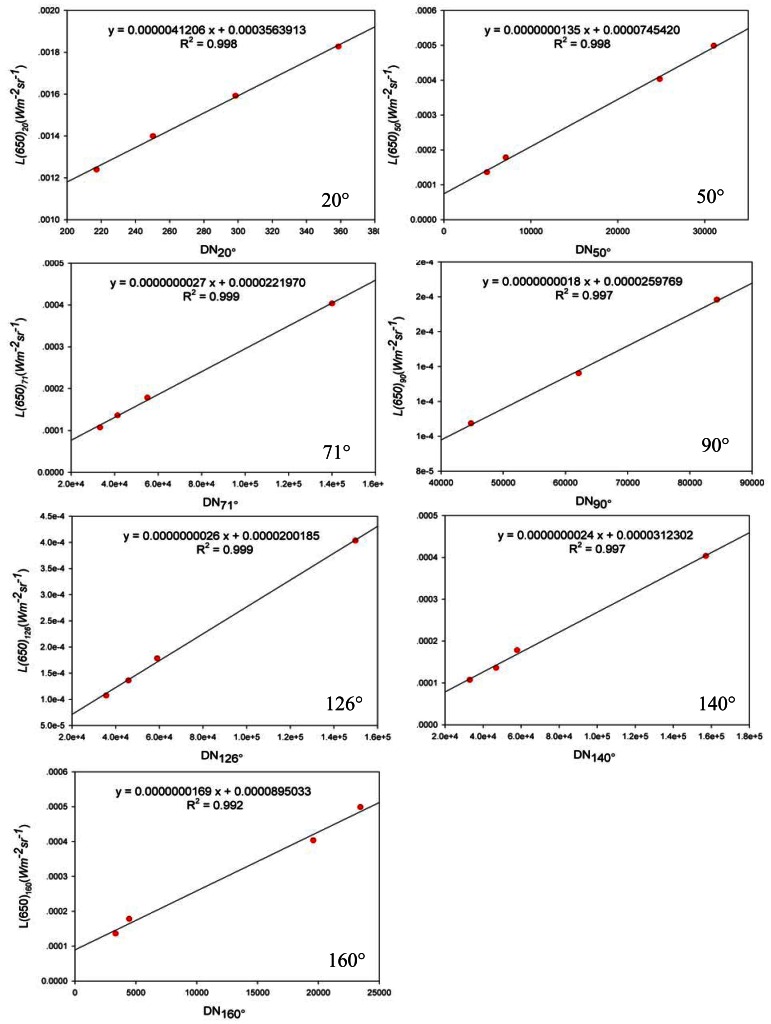
Calibration results of scattering light flux at seven angles: 20°, 50°, 71°, 90°, 126°, 140° and 160°.

We measured the response of each receiver to a Lambertian target by moving the target to different distances and recording the response at this distance. Using the Equation (14), the response functions of photoelectric signal *DN_p_* to radiance *L*(650) can be formed. Figure 9 shows the response functions of these scattering receivers. The correlation coefficients of these fitted straight lines for various scattering receivers are close to 1:

(15) $$L(650)=CDN_{P}$$

Coupled with the area of the collimator lens (*S_lens_*) and the solid angle Ω, the response function of the photoelectric signal *DN_p_* to the scattering flux Φ*_a_* can be obtained:

(16) $$\Phi_{a}=S_{\text{lens}}\Omega L(650)=S_{\text{lens}}\Omega CDN_{p}$$

where *C* is the calibration coefficient shown in Figure 9.

Because *S_lens_* and Ω are constant and can be calculated from optical and mechanical parameters, Equation (16) can be simplified as:

(17) $$\Phi_{a}=C_{s}DN_{p}$$

(18) $$C_{s}=CS_{\text{lens}}\Omega$$

In Figure 9, DN_20°_, DN_50°_, DN_71°_, DN_90°_, DN_126°_, DN_140°_, and DN_160°_ respectively represent the photoelectric signal *DN_p_* in different angles, and L(650)_20_, L(650)_50_, L(650)_71_, L(650)_90_, L(650)_126_, L(650)_140_, L(650)_160_ respectively represent the radiance received by different scattering angles.

After all of the receivers have been calibrated, the frequency modulation device in Figure 8 is moved, and the 1,000 W Quartz halogen spotlighting tungsten lamp is replaced by the 650 nm light source. Then, using the scattering receiver at 140° as the light flux detector to measure the response of the receiver to the light source, in combination with the calibration result of the scattering light flux at 140°, the light source Φ*_i_*(0,0) can be determined.

The receivers are calibrated in the optical laboratory in the air. If the receivers are immersed in water, the solid-angle FOV and the reflectance and transmittance of the water-optics interface with respect to the air-optics interface are changed. All of these changes are accounted for through the multiplication immersion factors *F_i_*(*λ*):

(19) $$\Phi_{w}=DN_{p}F_{i}(\lambda )C_{s}$$

In Equation (19), Φ*_w_* is the light flux received by the receiver in seawater, and *DN_p_* is the photoelectric signal of each receiver in seawater.

The determination of the immersion factor for the receiver is theoretically addressed by Austin [34], that is:

(20) $$F_{i}(\lambda )=\frac{n_{w}(\lambda ){\left(n_{w}(\lambda )+n_{g}(\lambda )\right)}^{2}}{{\left(1+n_{g}(\lambda )\right)}^{2}}$$

where *n_w_* is the wavelength-dependent refractive index of seawater and *n_g_* is the corresponding index of the glass window of the sensor. *n_w_* can be approximated by an empirical fit, according to Austin and Halikas [35]:

(21) $$n_{w}(\lambda )=1.325147+\frac{6.6096}{\lambda -137.1924}$$

The windows of the receivers consist of synthetic quartz glass, and *n_g_* at 650 nm is 1.45640. The immersion factor *F_i_*(*λ*) is equal to 1.731610 according to Equations (20) and (21), with a given *n_g_* and *λ*(*nm*).

According to Equation (7), we can conclude that the influence of immersion factor *F_i_*(*λ*) on *β*(*ψ*) is mainly through changing the attenuation coefficient *c*(*λ*). *c*(*λ*) must be calibrated by *F_i_*(*λ*) if the receivers are immersed in water.

The attenuation calibration test was performed in a clean 200 L (0.5 m high, 1.0 m wide, and 0.4 m deep) black water tank in an optics laboratory. A total of 120 L of high-purity water (resistivity: 18.2 MΩ) was poured into this water tank. At the beginning of the calibration test, the exterior of this VSF instrument was washed thoroughly with detergent, water and pure water, and the optical windows were cleaned with ethanol and lens tissue. Then, all of the sensors of the VSF instrument were submerged in the tank to measure *DN_tb_*, *DN_tb−d_*, *DN_ib_* and *DN_ib−d_*, which are the transmission receiver and light source signals and the dark current in a pure water standard, respectively. The attenuation calibration constant *C_t_* can be determined by the following equation after all of these parameters have been measured [36]:

(22) $$C_{t}=T_{b}(r_{d}+r_{s})\frac{DN_{ib}-DN_{ib-d}}{DN_{tb}-DN_{tb-d}}$$

where *T_b_*(*r_d_* + *r_s_*) is the transmittance of the pure water standard, *T_b_*(*r_d_* + *r_s_*) = exp[(−*c_w_*)(*r_d_* + *r_s_*)], and *c_w_* is the attenuation of the pure water standard.

After the calibration constant *c* is obtained, it is easy to calculate the attenuation of the water:

(23) $$c=\frac{1}{\left(r_{d}+r_{s}\right)}\ln\left\{\frac{\left[DN_{ib}-DN_{ib-d}\right]}{\left[DN_{tb}-DN_{tb-d}\right]}\frac{\left[DN_{tm}-DN_{tm-d}\right]}{\left[DN_{im}-DN_{im-d}\right]}\right\}+c_{w}$$

where *DN_tm_*, *DN_im_*, *DN_tm−d_*, and *DN_im−d_*, are the transmission detector and light source signals and dark current in the measurement water, respectively.

**Figure 10.**
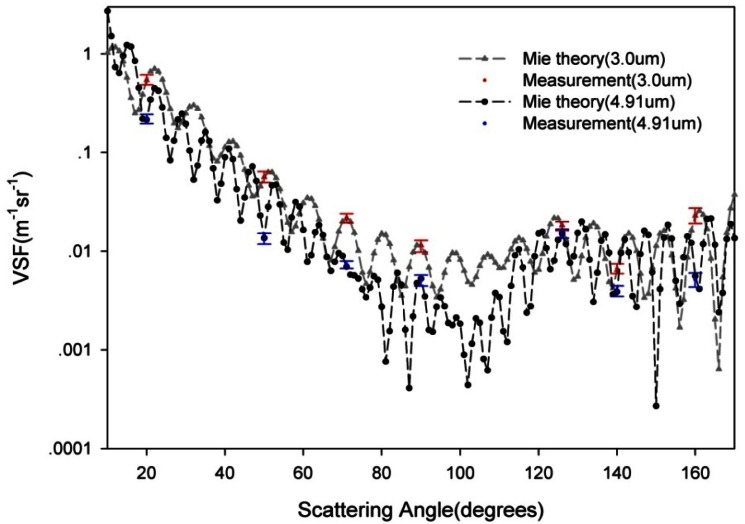
Comparison of theoretical Mie calculations and direct measurements of the VSF of polystyrene beads with different diameters (3 μm and 4.91 μm).

To estimate the accuracy of the VSF and the attenuation coefficient measured by this instrument, a series of comparative experiments on polystyrene beads (National Research Center for Certified Reference Material (NRCCRMS)) were performed in the water tank in the laboratory. These particles are monodisperse beads of precisely known size and refractive index. The observations of the VSF of these beads compared well to theoretical Mie calculations (Figure 10). For particles with two different central diameters (3.0 μm and 4.91 μm) and same refractive index (1.615), the theoretical Mie values and laboratory determination can be seen in Figure 10.

The expression for the relative error *δ*(*ψ*), which is a relative root mean square error at *ψ* is the following:

(24) $$\delta (\psi )=\sqrt{\frac{1}{N_{m}}\sum_{i=1}^{N_{m}}{\left(\frac{\beta^{i}(\psi )-\beta^{\text{Mie}}(\psi )}{\beta^{i}(\psi )}\right)}^{2}}$$

where *N_m_* is the number of VSF measurements (*N_m_* = 17 for 3.0 μm and *N_m_*=19 for 4.91 μm), *β^i^*(*ψ*) and *β^Mie^*(*ψ*) are respectfully a measurement and a Mie calculation. The average relative error *δ_average_*, that characterizes the total performance of the measurement is the root mean square value of *δ*(*ψ_j_*):

(25) $$\delta_{\text{aveage}}=\sqrt{\frac{1}{M}\sum_{j=1}^{M}\delta {\left(\psi_{j}\right)}^{2}}$$

where M = 7 is the number of scattering angles in our measurement.

The relative error *δ*(*ψ*) for the different angle and average relative error *δ_average_* for all seven scattering angles are presented in Table 1.

As we can see in Figure 10 and Table 1, for these experiments, the average error *δ_average_* is less than 0.12.

## 3. *In Situ* Measurement and Data Analysis

*In situ* experiments were performed in Daya Bay in the South China Sea on 26 July 2010. All *in situ* profile measurements were carried out in cruises on a 30 ton fishing-boat, using a manual winch lower and raising the VSF instrument through the water. The measurements data are recorded *in situ* continuously providing detailed VSF and depth information of the water column being tested. To investigate the variation of the VSF with angle, depth and space, the experiments were performed in areas with various water qualities, including the shallow (approximately 5.2 m deep) sea water breeding area of the Dongshan quay of Nanao (station A, inshore, 22°56′N, 114°52′E, near the National Field Station of the Marine Ecosystem at Daya Bay, Shenzhen) and the relatively deeper (approximately 10 m) sea water offshore Daya Bay (station B, 22°58′N, 114°54′E, near the Daya Bay Nuclear Power Station, and the water types (*i.e.*, attenuation coefficient) are similar to those of San Diego Harbor [23]).

**Figure 11.**
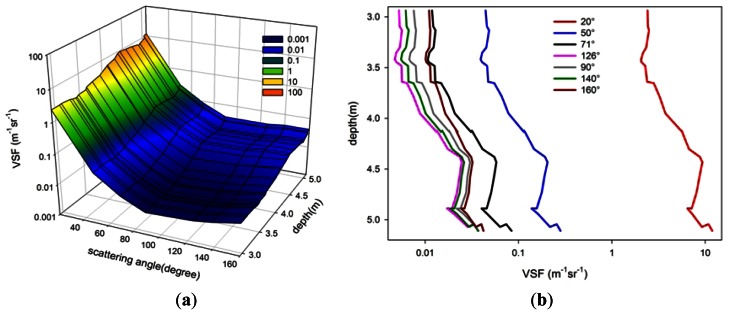
The distribution of the profile and the angle of the VSF (station A, inshore, 22°56′N, 114°52′E). (**a**) shows the three-dimensional distribution of the VSF with profile and angle; (**b**) shows the distribution characteristics of the VSF with profile at different angles.

**Figure 12.**
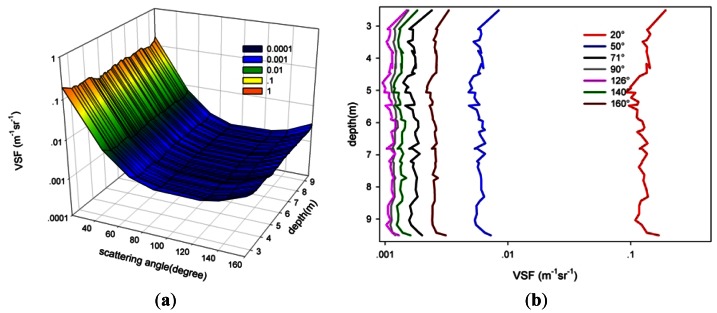
The distribution of the profile and the angle of the VSF (station B, 22°58′N, 114°54′E). (**a**) shows the three-dimensional distribution of the VSF with profile and angle; (**b**) shows the distribution characteristics of the VSF with profile at different angles.

Figure 11 shows the variation characteristics of the VSF with angle and depth in the shallow sea water breeding area of the Dongshan quay of Nanao (station A), while Figure 12 shows the variation characteristics of the VSF with angle and depth in the relatively deeper sea water offshore Daya Bay (station B). Figures 11(a) and 12(a) show the three-dimensional distribution of the VSF with profile and angle, and Figures 11(b) and 12(b) show the distribution characteristics of the VSF with profile at various angles.

The following points are easily seen:

1. Relative to the deeper sea water offshore Daya Bay (station B), the VSF of the shallow sea water breeding area of the Dongshan quay of Nanao (station A) varies obviously with depth, as shown in Figure 11. The VSF increases with depths between 3 m and 4.5 m, while it decreases with depths from 4.5 m to 5 m. It is worth noting that the maximum measurement depth is the seafloor. When the instrument contacts the seafloor, the sediment and some particulates could be stirred up, the VSF values of the water near the seafloor could increase, and the maximum at the seafloor might be reached.
2. In the same direction, the VSF of the inshore waters is far greater than that of the offshore waters. This result may be due to the contributions of the silt injection from the seacoast and plankton and other suspended particles, such as the excreta of creatures, all of which will affect the inherent optical properties of the inshore water.
3. In Daya Bay, the minimum of the VSF of the inshore waters is far greater than that of the offshore waters. This result may be due to the contributions of the silt injection from the seacoast and the plankton and other suspended particles, such as the excreta of creatures, all of which will affect the inherent optical properties of inshore water.

To examine the VSF shape variability in different directions (20° to 160°), the *β_ρ_*(*ψ*) measurements were normalized to *b_ρ_* (*b_ρ_* was synchronously measured *in situ* by a WETLabs AC-9) to obtain the particulate scattering phase functions in different directions and the backscattering fraction B_b_:

(26) $${\tilde{\beta }}_{p}(\psi )=\beta_{p}(\psi )/b_{p}$$

(27) $$B_{b}=\frac{b_{bp}}{b_{p}}$$

(28) $$b_{bp}=\chi_{p}(140)(\beta (140)-\beta_{w}(140))$$

*β_w_*(140) is obtained from the relationship [37]:

*χ_p_*(140) = 1.18*sr* [38,39]:

(29) $$\beta_{w}(\psi )=1.38{\left(650/500\right)}^{-432}(1+0.3S/37)10^{-4}(1+\cos^{2}(\psi ))(1-\delta )/(1+\delta )$$

where *δ* is depolarization ratio and *S* is salinity.

**Figure 13.**
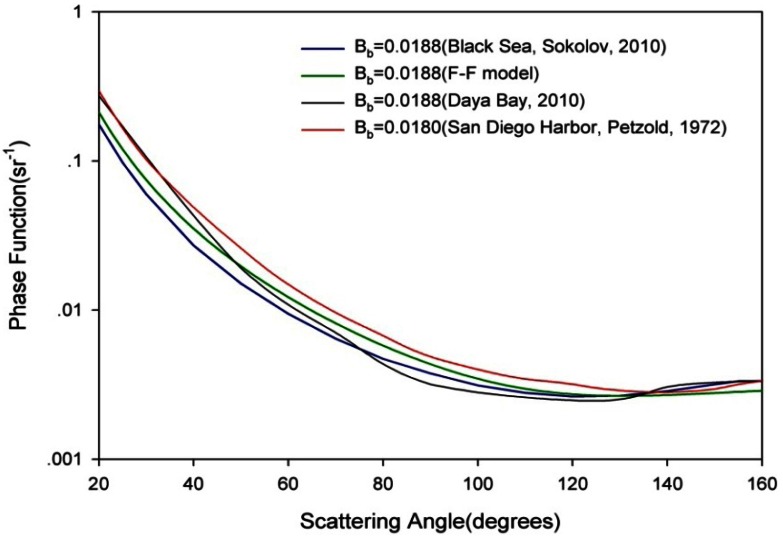
Comparison of Daya Bay 2010 phase function with phase function derived from Petzold measurements in San Diego Harbor [23], Sokolov measurements in Black Sea [4], and the Fournier-Forand (F-F) analytical phase functions. B_b_ for these phase functions are about 0.018.

To characterize our data set, we compared our phase function with FF theoretical functions [40], Sokolov measurements [4], and Petzold measurements [23] (Figure 13).

As we can see in Figure 13, the shapes of all scattering phase functions are in general similar; nevertheless some differences can also be noticed in Figure 13. The phase functions near 90° are below what was estimated from Fournier-Forand functions or Petzold measurements in San Diego Harbor or Sokolov measurements in the Black Sea. There is also a hump near 150° that agrees well with the Black Sea Sokolov measurements. The scattering phase function shapes near 90° or 150° are probably mainly due to the optical properties of the particles encountered in Daya Bay since these phenomena were not observed during the laboratory calibration beads experiments. These discrepancies could mainly due to high proportion of suspended calcium carbonate mineral-like particles with high refractive index in Daya Bay. Mie calculations of VSF for particles (for example refractive index greater than 1.55) showing a prevailing composition of highly refractive particles were performed and confirmed that such type of particles can lead to these occurrence; in addition, low resolutions of the angles inevitably also lost some information VSF.

## 4. Conclusions

The volume scattering function (VSF) is an important inherent optical property of water, and all inherent optical properties of water can be derived according to the VSF and the absorption coefficient. A new VSF profile measurement instrument, which is based on an array synchronization detection structure, was developed. The instrument is mainly used for synchronously measuring the transmission at 0° and the VSF in seven different angles: 20°, 50°, 71°, 90°, 126°, 140° and 160°. The calibration method used for the VSF involves measuring the response of the scattering receiver to a Lambertian target and an approximation of the scattering volume. The measurement error of the VSF measurement instrument has been estimated in the laboratory based on the Mie theory, and the average error is less than 12%. The instrument was applied to measure and analyze the variation characteristics of that the VSF with angle, depth and water quality in the Daya Bay for the first time. The *in situ* results indicated the VSF of the inshore waters is far greater than that of the offshore waters. This result may be due to the contributions of the silt injection from the seacoast and plankton and other suspended particles and we also found that the phase functions proposed by Fournier-Forand, measured by Petzold in San Diego Harbor and Sokolov in the Black Sea do not fit with our measurement in Daya. These discrepancies could mainly due to the high proportion of suspended calcium carbonate mineral-like particles with high refractive index.

The VSF instrument presented in this paper can quickly and synchronously measure the VSF and attenuation coefficient *in situ* (profile depth up to 200 m and acquisition frequency is 6 Hz), and the features of low power consumption and high resource utilization make it perfect for long time *in situ* research. Based on the instrument, in order to more comprehensive study the variation of VSF with angle and wavelength, and to accurately obtain scattering coefficients/backscattering coefficients by VSF integration, a lot of improvements will be made on the structure and function of this instrument prototype like adding operation wavelengths and angles to realize multi-wavelength, general-angle scattering measurements *in situ*.

## Supplementary Material



## Figures and Tables

**Table 1.** The relative error *δ*(*ψ*) and the average error *δ_average_*.

*ψ*°	**20**	**50**	**71**	**90**	**126**	**140**	**160**	**Averaged**

**Diameter**, μm	*δ*(20)	*δ*(50)	*δ*(71)	*δ*(90)	*δ*(126)	*δ*(140)	*δ*(160)	*δ_average_*
3.0 μm	0.117	0.126	0.102	0.12	0.101	0.119	0.145	0.119
4.91 μm	0.109	0.121	0.099	0.115	0.099	0.123	0.140	0.115
