# Supplementary material for: An Instrument for In Situ Measuring the Volume Scattering Function of Water: Design, Calibration and Primary Experiments
Source: Sensors (Basel). 2012 Apr 10;12(4):4514–33. doi: 10.3390/s120404514 (PMC3355425; doi:10.3390/s120404514)
Supplement: Supplementary file 1 [file sensors-12-04514-s001.pdf]

Supplementary Information

An Instrument for *In Situ* Measuring the Volume Scattering Function of Water: Design, Calibration and Primary Experiments

Cai Li [1], Wenxi Cao [1,*], Jing Yu [2], Tiancun Ke [1], Guixin Lu [1], Yuezhong Yang [1] and Chaoying Guo [1]

[1] State Key Laboratory of Oceanography in the Tropics, South China Sea Institute of Oceanology, Chinese Academy of Sciences, Guangzhou 510301, China; E-Mails: liclaire@scsio.ac.cn (C.L.); ketian@scsio.ac.cn (T.K.); luguixin@scsio.ac.cn (G.L.); wuli@scsio.ac.cn (Y.Y.); guochaoying@scsio.ac.cn (C.G.)

[2] South China Sea Fisheries Research Institute, Chinese Academy of Fishery Sciences, Guangzhou 510300, China; E-Mail: yj_scs@163.com

***** Author to whom correspondence should be addressed; E-Mail: wxcao@scsio.ac.cn; Tel.: +86-20-8902-3168; Fax: +86-20-8902-3167.

Received: 29 February 2012; in revised form: 29 March 2012 / Accepted: 30 March 2012 / Published: 10 April 2012

---

1. The Derivation of Scattering Volume

The scattering volume varies with the detection direction of the scattering light: it is smallest at 90 ° and becomes larger when approaching forward 0 ° or backward 180 °. This is a complex process. Here, we illustrate the calculation method of the scattering volume based on the design of the optical and mechanical structures. In Figures 1 and 2, rs is the distance from the center of the scattering volume to the light source, rd is the distance from the scattering receiver to the center of the scattering volume, θ is angle of half field-of view (FOV) of the scattering receiver, D is the diameter of the light source aperture, and ψ is the scattering angle. Because the beam divergence is less than 0.02 mrad, rs and rd are 25 cm, so the divergence of the light source at the distance of rs can be overlooked and thought of as an even-diameter cylindrical light in the light travel range. The light source distribution is homogeneous, and the receiving FOV at each point of the scattering receiver is equal. In addition, the diameter of the light source is less than the diameter of each scattering receiver. Based on single scattering approximation, the relationship between the scattering volume (including $V(90°)$ and $V(\psi)$) and the optical and geometrical structures of the instrument can be deducted and computed.

In Figure 2, the scattering volume can be approximated as a frustum of ellipsoid shape, and the top area of it is S1, bottom area of it is S2, and the height of it is h. In Figure 2, $r1$ is the distance between O and E, $r2$ is the distance between O and F, $D1$a is the distance between D and F, $D1$b is the distance between F and C, $D2$a is the distance between A and E, $D2$b is the distance between E and B, θ is angle of half field-of view, ψ is scattering angle, $R1$ is long axis of top ellipse (S1), $R2$ is long axis of bottom ellipse (S2), r_d is the distance between O and G.

Figure 1. Optical structure of the measuring instrument (light source and detector).

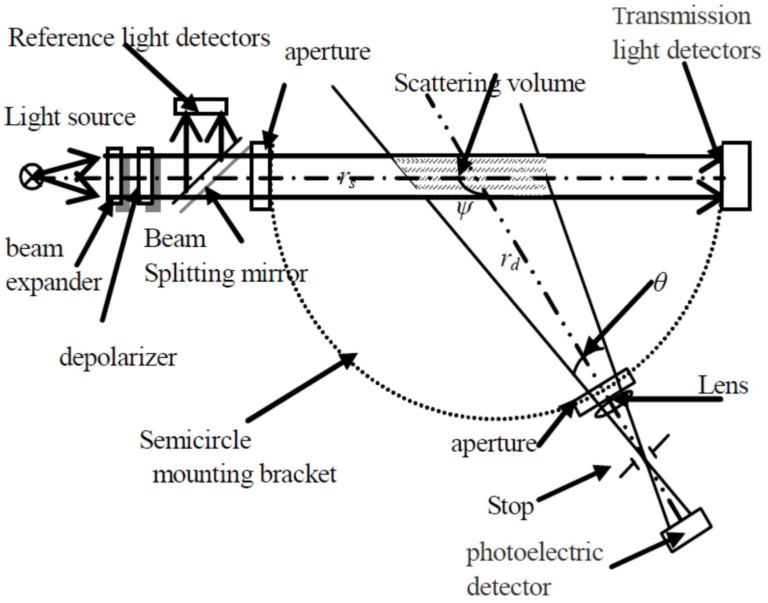

Figure 2. Scattering volume change with scattering angle.

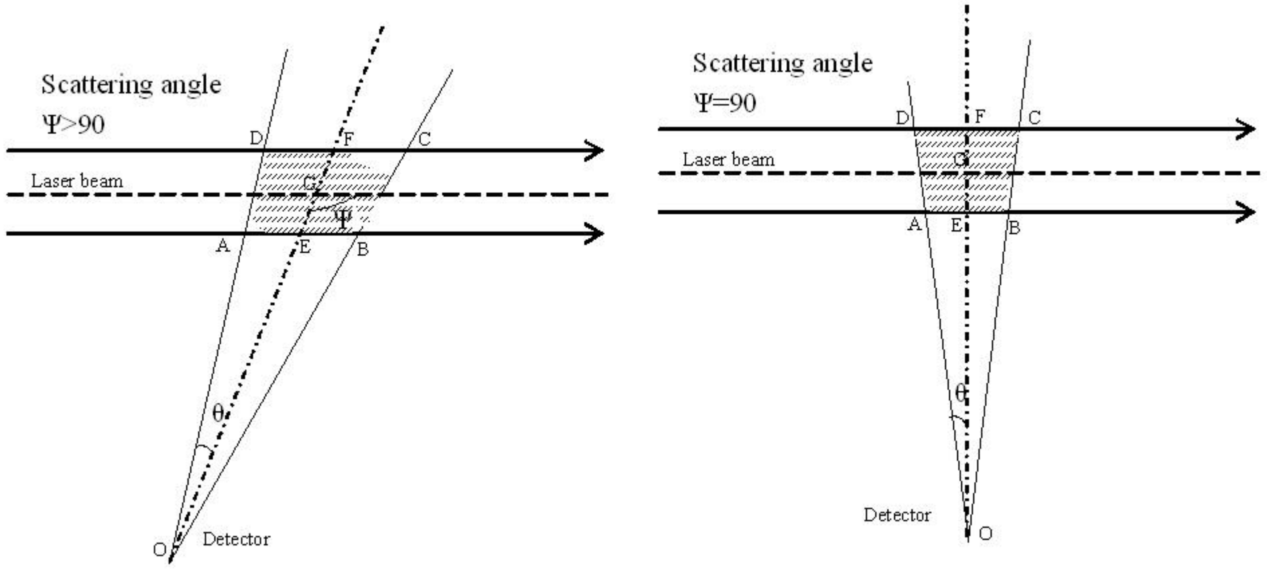

From Figure 2, because the diameter of the light source is less than the diameter of each scattering receiver, the short axis of S1 and S2 are D/2, and h = D.

The scattering volume calculated in two cases:

A: *V*(90°) **scattering angle ψ = 90 °**

$$r1 = r_d + \frac{D}{2}$$

$$r2 = r_d - \frac{D}{2}$$

$$R1 = r1 tg\theta$$

$$R2 = r2 tg\theta$$

The volume of the frustum of the ellipsoid shape is

$$V(90) = \frac{1}{3}h(S1 + \sqrt{S1S2} + S2)$$

$$V(90) = \frac{1}{3}\pi h(\frac{D}{2}R1 + \frac{D}{2}\sqrt{R1R2} + \frac{D}{2}R2) = \frac{\pi D}{6}h(R1 + \sqrt{R1R2} + R2)$$
$$= \frac{\pi D^2}{6}tg\theta\left(2r_d + \sqrt{r_d^2 - \left(\frac{D}{2}\right)^2}\right) \quad (1)$$

B: $V(\psi)$ **scattering angle ψ<>90** °(*i.e.*, 180 °> ψ > 90 °or 0 °< ψ <90 °)

$$r1 = r_d + \frac{D}{2\sin\psi}$$

$$r2 = r_d - \frac{D}{2\sin\psi}$$

$$\frac{r1}{\sin(\psi+\theta)} = \frac{D1a}{\sin\theta} \Rightarrow D1a = \frac{r1\sin\theta}{\sin(\psi+\theta)}$$

$$\frac{r1}{\sin(\psi-\theta)} = \frac{D1b}{\sin\theta} \Rightarrow D1b = \frac{r1\sin\theta}{\sin(\psi-\theta)}$$

$$\frac{r2}{\sin(\psi+\theta)} = \frac{D2a}{\sin\theta} \Rightarrow D2a = \frac{r2\sin\theta}{\sin(\psi+\theta)}$$

$$\frac{r2}{\sin(\psi-\theta)} = \frac{D2b}{\sin\theta} \Rightarrow D2b = \frac{r2\sin\theta}{\sin(\psi-\theta)}$$

R1 and R2 can be calculated from D1a, D1b, D2a, D2b:

$$R1 = (D1a + D1b)/2 = \frac{r1\sin\theta}{2}A$$

$$R2 = (D2a + D2b)/2 = \frac{r2\sin\theta}{2}A$$

Where $A = \frac{1}{\sin(\psi+\theta)} + \frac{1}{\sin(\psi-\theta)} = \frac{2\sin\psi\sin\theta}{\sin^2\psi - \sin^2\theta}$

The volume of the frustum of the ellipsoid shape is:

$$V(\psi) = \frac{1}{3}h(S1 + \sqrt{S1S2} + S2)$$

$$
\begin{aligned}
V(\psi) &= \frac{1}{3}\pi h(\frac{D}{2}R1+\frac{D}{2}\sqrt{R1R2}+\frac{D}{2}R2)=\frac{\pi D}{6}h(R1+\sqrt{R1R2}+R2)\\
&= \frac{\pi D}{6}h(\frac{r1\sin\theta}{2}A+\frac{\sin\theta}{2}A\sqrt{r1r2}+\frac{r2\sin\theta}{2}A)\\
&= \frac{\pi D\sin\theta}{12}Ah(r1+\sqrt{r1r2}+r2)\\
&= \frac{\pi D\sin\theta}{12}Ah(2r_d+\sqrt{r_d{}^2-\left(\frac{D}{2\sin\psi}\right)^2})\\
&= \frac{\pi D^2\sin\theta}{6}\frac{\sin\psi\cos\theta}{\sin^2\psi-\sin^2\theta}\left(2r_d+\sqrt{r_d{}^2-\left(\frac{D}{2\sin\psi}\right)^2}\right)\\
&= \frac{\pi D^2}{6}tg\theta\frac{\sin\psi\cos^2\theta}{\sin^2\psi-\sin^2\theta}\left(2r_d+\sqrt{r_d{}^2-\left(\frac{D}{2\sin\psi}\right)^2}\right) \qquad (2)
\end{aligned}
$$

© 2012 by the authors; licensee MDPI, Basel, Switzerland. This article is an open access article distributed under the terms and conditions of the Creative Commons Attribution license (http://creativecommons.org/licenses/by/3.0/).
